# Pediatric Essential Thrombocythemia: A Case of a JAK2-Mutated Adolescent With Microvascular Symptoms

**DOI:** 10.7759/cureus.99370

**Published:** 2025-12-16

**Authors:** Madalena Fonseca, Ana Cristóvão Ferreira, Carolina Amaro Gonçalves, Anabela Ferrão

**Affiliations:** 1 Pediatrics, Hospital Santa Maria, Unidade Local de Saúde Santa Maria, Lisbon, PRT; 2 Pediatric Hematology Unit, Hospital Santa Maria, Unidade Local de Saúde Santa Maria, Lisbon, PRT

**Keywords:** acquired von willebrand disease, erythromelalgia, essential thrombocythemia, hydroxyurea, jak2 v617f mutation

## Abstract

Thrombocytosis, defined as platelet counts >450 × 10⁹/L, is frequent in the pediatric population and usually secondary to inflammatory conditions or iron deficiency. Essential thrombocythemia (ET), a Philadelphia chromosome-negative myeloproliferative neoplasm, is exceptionally rare in childhood. Pediatric ET often follows an indolent course but carries risks of thrombotic and hemorrhagic events, as well as late progression to myelofibrosis or leukemia. We report the case of a 14-year-old girl presenting with recurrent acral edema, erythema alternating with cyanosis, burning pain, paresthesia, and headaches. Physical examination was unremarkable. Initial suspicion of Raynaud’s phenomenon was excluded by nailfold capillaroscopy. Laboratory studies revealed persistent thrombocytosis with platelets over 1,092 × 10⁹/L. Secondary causes were excluded. Bone marrow biopsy revealed megakaryocytic hyperplasia with hyperlobulated megakaryocytes, abdominal ultrasound revealed hepatosplenomegaly, and molecular testing identified a *JAK2 V617F* mutation, confirming ET. She was initially treated with low-dose acetylsalicylic acid, with partial improvement, but microvascular symptoms persisted, and platelet counts remained >1,000 × 10⁹/L. Hydroxyurea was initiated, leading to progressive platelet reduction and marked clinical benefit. Over three years of follow-up, the patient remained clinically stable, without adverse effects or leukemic transformation. This case illustrates the rarity and diagnostic complexity of pediatric ET, which requires exclusion of reactive causes, bone marrow evaluation, and molecular testing. Management remains particularly challenging due to the absence of pediatric-specific guidelines, with current approaches being largely derived from adult protocols. Cytoreductive therapy may be indicated in cases with extreme thrombocytosis or refractory symptoms, and long-term follow-up is crucial to monitor disease evolution and treatment outcomes. This case highlights the need for multicenter studies and international registries to have pediatric-specific evidence that can better inform diagnostic and therapeutic strategies.

## Introduction

Thrombocytosis, defined as a platelet count >450 × 10⁹/L, is relatively common in children and often detected incidentally [1]. Most cases are transient and reactive, secondary to infectious/inflammatory conditions or iron deficiency [2]. Primary thrombocytosis, particularly essential thrombocythemia (ET), is exceedingly rare, with an estimated incidence of 1 per 10,000,000 children under 14 years of age [3]. Pediatric ET typically follows an indolent course but may carry thrombotic or hemorrhagic risks and rarely progress to myelofibrosis or leukemia [4]. Pediatric management must be carefully individualized, balancing long-term treatment risks against potential vascular complications. Here, we report a case of *JAK2*-positive ET in an adolescent girl that illustrates the diagnostic and therapeutic considerations unique to the pediatric setting.

## Case presentation

A 14-year-and-10-month-old female adolescent was referred to the Pediatric Hematology Unit of a tertiary hospital for recurrent episodes of acral edema, erythema alternating with cyanosis, burning pain, paresthesia, and recurrent headaches. Physical examination was unremarkable, with no skin lesions, digital ulcers, or organomegaly. There was no history of thrombosis, bleeding, or anemia. She denied medication use and had no family history of similar symptoms, including her asymptomatic homozygotic twin sister.

She was initially referred to the Rheumatology Department due to a clinical suspicion of Raynaud’s phenomenon, which was later excluded based on nailfold capillaroscopy findings. Autoimmune studies revealed antinuclear antibodies, weakly positive, while the remaining tests were negative.

Laboratory studies demonstrated marked thrombocytosis (platelets: 1,092 × 10⁹/L) with otherwise normal hematologic parameters. A review of prior results revealed persistent thrombocytosis over the previous two years (505-589 × 10⁹/L) that had not been investigated further. The vascular manifestations were interpreted as erythromelalgia secondary to thrombocytosis of initially unclear etiology. Secondary causes were excluded, as presented in Table 1.

**Table 1:** Initial investigation findings. PT = prothrombin time; APTT = activated partial thromboplastin time; VWF = von Willebrand factor; anti-HIV 1/2 = anti-human immunodeficiency virus 1/2; HBsAg = hepatitis B surface antigen; HCV = hepatitis C virus; ANA = antinuclear antibodies; Anti-SSA = anti-Sjögren’s syndrome A antibody; Anti-SSB = anti-Sjögren’s syndrome B antibody; Anti-SM = anti-Smith antibody

Category	Test	Results	Reference range
Hematology	Hemoglobin	14.4 g/dL	12.0–15.3 g/dL
	Mean corpuscular volume	79.8 fL	80–100 fL
	Hematocrit	43.6%	36–46%
	Total leucocytes	7.1 × 10^9^/L	4.0–11.0 × 10^9^/L
	Platelets	1,092 × 10^9^/L	150–450 × 10^9^/L
	Blood smear	No blasts or abnormal cells	
	Erythrocyte sedimentation rate	2 mm	<12 mm/1^st^ hour
Coagulation studies	PT	12.7 seconds	Control: 11.9 seconds
	APTT	33 seconds	Control: 39 seconds
	Factor VIII	81%	50-150%
	VWF antigen	71%	66.1–176.3%
	VWF ristocetin cofactor activity assay	42%	60.8–229.8%
Iron metabolism	Ferritin	26.3 ng/mL	<12 mg/dL
Infectious	C-reactive protein	0.05 mg/dL	<0.5 mg/dL
	Anti-HIV 1/2	Negative	-
	HBsAg	Negative	-
	Anti-HCV	Negative	-
Autoimmunity	ANA	1/160	-
	Anti-SSA	Negative	-
	Anti-SSB	Negative	-
	Anti-SM	Negative	-

Abdominal ultrasonography revealed mild hepatomegaly (liver bipolar diameter: 160 mm) and splenomegaly (spleen bipolar diameter: 136 mm), with preserved parenchymal structure and no additional abnormalities. Bone marrow biopsy revealed hypercellularity, primarily due to the megakaryocytic lineage, with maturation preserved in all three lineages. The megakaryocytic lineage demonstrated numerous enlarged, hyperlobulated megakaryocytes arranged in clusters, without significant reticulin acentuations. Findings were consistent with ET.

Genetic testing identified a somatic *JAK2 V617F* mutation (allelic frequency of 17%), consistent with essential thrombocythemia; no other mutations were detected, including no detectable *BCR::ABL1* gene. A diagnosis of *JAK2*-positive ET was established.

**Figure 1:**
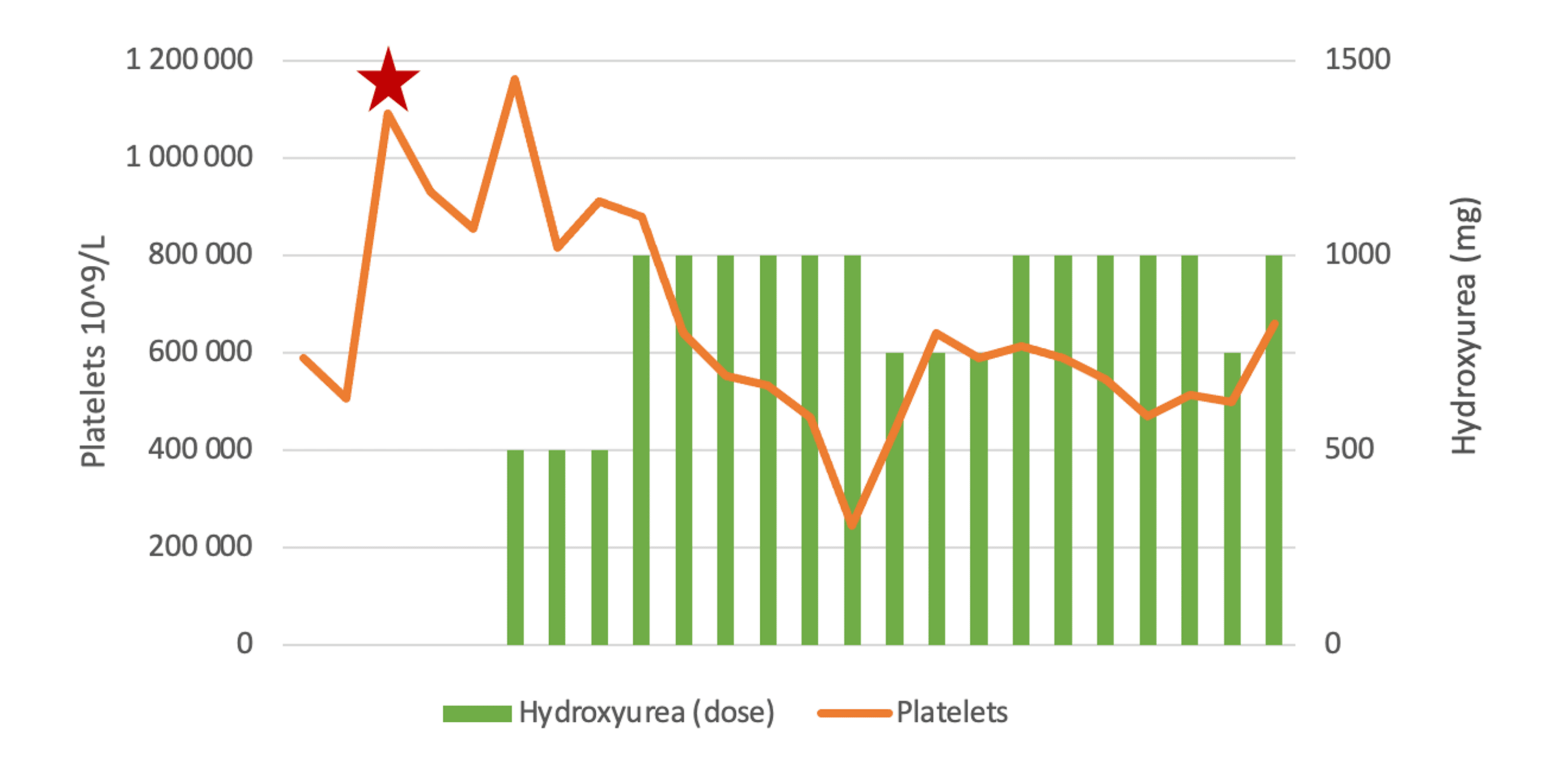
Evolution of platelet counts and hydroxyurea dosing. The star points to the beginning of the follow-up.

Low-dose acetylsalicylic acid (75 mg/day) was initiated, resulting in partial improvement in headaches and edema, although paresthesia persisted. Because platelet counts remained >1,000 × 10⁹/L with ongoing microvascular symptoms, hydroxyurea was introduced (initially 500 mg/day and subsequently increased to 1,000 mg/day), and aspirin was discontinued. Figure 1 summarizes the evolution of platelet counts and hydroxyurea dosing. As shown, and given the favorable hematologic and symptomatic response at 1,000 mg/day, several attempts were made to taper the hydroxyurea dose to minimize potential adverse effects. However, each dose reduction led to a rise in platelet counts accompanied by mild recurrence of microvascular symptoms. Consequently, a maintenance dose of 1,000 mg/day was required to achieve sustained disease control.

The patient has been followed for three years, with sustained clinical benefit and no treatment-related side effects. During follow-up, she developed easy bruising with no other signs of bleeding tendency. Laboratory evaluation revealed reduced von Willebrand factor activity, consistent with acquired von Willebrand disease, presumed secondary to extreme thrombocytosis resulting in shear-induced proteolysis of von Willebrand factor multimers. She continues to be monitored every three months, with complete blood counts, assessment of microvascular symptoms, and surveillance for hydroxyurea-related adverse effects.

## Discussion

Thrombocytosis is defined as a platelet count exceeding 450 × 10⁹/L (1,20) [1]. It can be stratified into mild (450-700 × 10⁹/L), moderate (700-900 × 10⁹/L), and severe (>900 × 10⁹/L) forms, while counts above 1,000 × 10⁹/L are considered extreme thrombocytosis [5]. Extreme thrombocytosis is uncommon in children, documented in approximately 0.5% of hospitalized pediatric patients [1].

Thrombocytosis can be classified as primary (essential) or secondary (reactive) [6]. ET is a chronic myeloproliferative neoplasm (MPN) characterized by sustained, uncontrolled platelet production as the predominant hematologic abnormality [5]. ET represents one of the classic *BCR::ABL*-negative MPNs. Other Philadelphia chromosome-negative MPNs include polycythemia vera and primary myelofibrosis [2]. Chronic myeloid leukemia, a *BCR::ABL*-positive MPN, may occasionally present with thrombocytosis but is distinguished by its molecular hallmark [2].

Secondary thrombocytosis is far more common in children, affecting approximately 5-15% of hospitalized patients [1,2,5]. It arises from reactive stimulation of megakaryopoiesis due to a variety of hematological and non-hematological conditions. Most children exhibit mild platelet elevations, although transient extreme thrombocytosis may occasionally occur [1]. True ET in childhood, by contrast, is exceedingly rare [1,6].

The etiology of reactive thrombocytosis is diverse (Table 2). Infectious diseases represent the most frequent cause, particularly in young children under one year of age, in whom virtually any infection may trigger significant thrombocytosis [1,2,6]. Inflammatory disorders such as Kawasaki disease, juvenile idiopathic arthritis, and inflammatory bowel disease are also well-recognized contributors [1]. Reactive thrombocytosis is largely mediated by the upregulation of thrombopoietin (TPO), as inflammation enhances hepatic TPO mRNA expression [2]. Circulating TPO concentrations have been shown to correlate with C-reactive protein levels, further supporting the link between systemic inflammation and thrombopoietin-driven thrombocytosis [2].

**Table 2:** Causes of secondary (reactive) thrombocytosis in children.

Category	Causes
Infectious	Viral infections; bacterial infections (acute and chronic)
Inflammatory	Kawasaki disease; rheumatoid arthritis; inflammatory bowel disease; connective tissue disorders; celiac disease
Pharmacological	Corticosteroids; vincristine; tretinoin; epinephrine; beta-lactam antibiotics; miconazole; haloperidol; low-molecular-weight heparins; cocaine or maternal morphine exposure (in neonates)
Other causes	Iron deficiency anemia; nephritis/nephrotic syndrome; pancreatitis; trauma; malignancy; hyposplenism or post-splenectomy states

Iron deficiency is another common cause, partly attributed to the structural homology between erythropoietin and TPO, which may promote megakaryocyte proliferation in anemic states [2].

On the other hand, ET predominantly affects middle-aged adults, with an incidence of approximately 2 per 100,000 per year [6]. Pediatric ET is rare, with an estimated incidence of 1 per 10,000,000 children under 14 years of age, roughly 200 times lower than in adults [6,7].

The largest pediatric ET cohort, reported by Fu et al. (2025) in China, included 156 children aged 1-18 years (median age 13) [4]. A slight male predominance was observed (81 males), although this was not statistically significant (p > 0.05) [4]. These data underscore the rarity of pediatric ET and highlight the need for multicenter studies to better characterize its clinical and molecular features.

Currently, no pediatric-specific diagnostic criteria exist. Adult World Health Organization criteria (2022) are generally applied, which are summarized in Table 3 [8].

**Table 3:** Diagnostic criteria for essential thrombocythemia. Adapted from Arber et al. (2022) [18]. CML = chronic myeloid leukemia; MDS = myelodysplastic syndromes; PV = polycythemia vera; PMF = primary myelofibrosis

	Criteria
Major criteria	1. Platelet count ≥450 × 10^9^/L
	2. Bone marrow biopsy showing proliferation mainly of the megakaryocytic lineage, with increased numbers of enlarged, mature megakaryocytes with hyperlobulated staghorn-like nuclei, infrequently dense clusters; no significant increase or left shift in neutrophil granulopoiesis or erythropoiesis; no relevant bone marrow fibrosis
	3. Not meeting WHO criteria for *BCR::ABL1* positive CML, PV, PMF, MDS, or other myeloid neoplasms
	4. Presence of *JAK2*, *CALR*, or *MPL* mutation
Minor criteria	Presence of a clonal marker (e.g., abnormal karyotype) or absence of evidence for reactive thrombocytosis
ET diagnosis requires meeting all four major criteria or the first three major criteria and one of the minor criteria	

ET in adults typically harbors clonal driver mutations: *JAK2 V617F* in 50-60%, *CALR* in 15-24%, *MPL* in ~4%, with around 20% triple-negative cases [6,9,10]. In contrast, pediatric ET displays a much lower frequency of these canonical mutations [11]. In the Fu et al. cohort, only 31% of children had driver mutations; Randi et al. reported that approximately 75% of 89 pediatric cases were triple-negative [4,12].

Our patient carried the *JAK2 V617F* mutation, representing a minority of children with detectable clonal markers. This mutation is associated with higher symptom burden, including headaches, paresthesia, and gastrointestinal complaints [4]. The *JAK2* gene belongs to the Janus kinase (JAK) family of non-receptor tyrosine kinases and acts as a key signal transducer in hematopoietic cell proliferation [9]. The *JAK2 V617F* mutation is a constitutively active mutation in the pseudokinase domain that activates the JAK/STAT pathway, leading to uncontrolled cellular proliferation in the absence of normal cytokine signaling [2,9]. *CALR* and *MPL* mutations also converge on the JAK/STAT pathway, reinforcing this abnormal proliferative signaling [9].

Although driver mutations constitute the molecular hallmark of adult ET, they are detected far less frequently in pediatric cases, as stated before [11]. Moreover, bone marrow morphology in children does not always align with the expected features, and up to one-quarter of patients may show normal histology [9]. This means that a non-diagnostic biopsy should not exclude ET if clinical suspicion remains strong [9]. These differences not only distinguish pediatric ET from the adult disease but also challenge the direct application of adult-derived diagnostic criteria in children [5,9,12]. This reinforces the need for pediatric-specific diagnostic guidelines and ongoing longitudinal monitoring, as small clonal populations may become detectable over time [4].

Most pediatric ET cases are asymptomatic and detected incidentally, although microvascular symptoms such as headache or erythromelalgia may occur. Symptomatic patients usually present with microvascular or neurological complaints, including headache, dizziness, paresthesia, transient ischemic events, erythromelalgia, or visual disturbances [6]. Gastrointestinal symptoms (nausea, vomiting, abdominal pain) and organomegaly, particularly splenomegaly, are also relatively common [6].

From a clinical standpoint, our patient exemplifies the complexities of recognizing ET in childhood. She initially presented with recurrent vascular symptoms with edema, erythema alternating with cyanosis, paresthesia, and burning pain in the extremities that were initially misinterpreted as Raynaud’s phenomenon, leading to referral to a rheumatologist.

Pediatric ET generally carries a lower risk of vascular events compared with adult ET; however, thrombotic and hemorrhagic complications remain clinically relevant, with reported thrombotic rates ranging from 0% to 11% and major bleeding being rare [4,6,7,11]. When thromboses occur in children, they can be severe, particularly in the presence of *JAK2 V617F* mutations or hyperviscosity symptoms [4]. Data from the Fu et al. cohort of 156 children with ET shows that thrombotic events occurred in 5.8% of cases, with 2.6% occurring before diagnosis and 4.5% during follow-up (0.86 per 100 patient-years) [4].

Hemorrhagic complications remain uncommon but may arise with extreme thrombocytosis (>1,000 × 10⁹/L), which can lead to acquired von Willebrand disease (AvWd) [10]. In the same cohort, two children (1.3%) experienced major bleeding, one while on aspirin (von Willebrand factor activity: 56.9%) and one without antiplatelet therapy [4]. Therefore, current recommendations advise assessing von Willebrand factor antigen and function in patients with extreme thrombocytosis, and if AvWD is diagnosed, low-dose aspirin should be withheld [10].

In the absence of pediatric-specific guidelines, management is adapted from adult practice, with emphasis on individualized decision making [6,9,10]. The main goal of therapy is to prevent major vascular events while minimizing treatment-related side effects.

To achieve these goals, risk stratification is essential. In adults, risk is mainly based on age and history of thrombosis; however, this approach has not been validated in children, who generally have a lower risk of vascular complications and a longer disease course [2,12]. Therefore, pediatric-specific risk assessment remains an area of ongoing investigation.

Kucine et al. (2014) proposed a practical framework for pediatric patients [2]: (i) Asymptomatic children: observation is recommended. No immediate treatment. Blood counts should be monitored every three to six months [2]. (ii) Low-risk patients: include symptomatic children, hepatosplenomegaly, cardiac risk factors, or additional thrombophilia can be managed with low-dose aspirin (2-3 mg/kg/day, up to a maximum of 75 mg daily) [2,10]. Aspirin should be used cautiously in children younger than 12 years due to the risk of Reye syndrome, and it is contraindicated in patients with AvWD, extreme thrombocytosis (>1,500 × 10⁹/L), or previous major aspirin-associated bleeding [6,7,10]. (iii) High-risk patients: children with platelet counts >1,000 × 10⁹/L, prior history of thrombosis/bleeding, or failure of low-risk therapy, should be considered for cytoreductive treatment [2].

Cytoreductive therapy options include hydroxyurea, interferon-alpha (IFN-α), and anagrelide. As there is no consensus on first-line therapy in children, treatment decisions should be individualized in consultation with the patient and family [6,9,10].

Hydroxyurea is an S-phase antimetabolite that inhibits ribonucleotide reductase, slowing DNA synthesis. Side effects are generally mild and reversible, including bone marrow suppression, gastrointestinal symptoms, and skin changes. Although long-term leukemogenic risk remains debated, cumulative data from pediatric sickle cell disease cohorts are reassuring, supporting its safety in children [6,10,13].

IFN-α suppresses hematopoietic progenitor proliferation and inhibits TPO-induced megakaryocyte growth [13]. IFN-α effectively reduces platelet counts without increasing leukemogenic or teratogenic risk. However, its persistent side effects, including flu-like symptoms, neuropsychiatric effects, and subcutaneous administration, can limit adherence in children [2,13].

Anagrelide is an orally active platelet-lowering agent that inhibits megakaryocyte maturation and proplatelet formation. It is non-leukemogenic but has limited pediatric experience. In adults, it has been associated with higher rates of fibrotic transformation, hemorrhagic events, and arterial embolism compared with hydroxyurea [10,13]. Overall, treatment in pediatric ET should be individualized, balancing the risk of thrombosis, bleeding, medication side effects, and patient/family preferences [6].

Given the presence of microvascular symptoms, including erythromelalgia and recurrent headaches, and the absence of hemorrhagic diathesis, our patient was initially started on low-dose aspirin. However, symptoms persisted despite therapy, and platelet counts remained consistently above 1,000 × 10⁹/L. Hydroxyurea was subsequently initiated, selected due to our center’s extensive experience with this therapy in pediatric patients, including those with sickle cell disease, and after discussion with the patient regarding potential side effects and the importance of effective contraceptive measures. Following hydroxyurea initiation, the patient experienced a marked improvement in symptoms, with progressive reduction in platelet counts and no evidence, to date, of treatment-related adverse effects or leukemic transformation.

## Conclusions

This case highlights the diagnostic and therapeutic challenges of ET in the pediatric setting, showing that even with symptoms suggestive of the disease and persistent thrombocytosis over a two-year period, referral and recognition of clonal thrombocytosis were delayed. Accurate diagnosis ultimately allowed for structured monitoring and informed evaluation of treatment options for the patient’s microvascular symptoms. Therapeutic decisions must be individualized in symptomatic cases, balancing the risks and benefits of cytoreductive therapy. This case underscores the lack of pediatric-specific guidelines and the need for international databases and registries to harmonize diagnostic criteria, support clinical decision-making, and optimize long-term management.
